# Human Neutrophil Elastase and the Protein-Storm Axis: Reversible Synthetic Inhibitors in Inflammatory Disease

**DOI:** 10.3390/molecules31091441

**Published:** 2026-04-27

**Authors:** Simona Viglio, Maria Antonietta Grignano, Marilena Gregorini, Teresa Rampino, Giampiero Pietrocola, Paolo Iadarola

**Affiliations:** 1 Department of Molecular Medicine, University of Pavia, 27100 Pavia, Italy; simona.viglio@unipv.it (S.V.); giampiero.pietrocola@unipv.it (G.P.); 2 Research Department, IRCCS Policlinico San Matteo Foundation, 27100 Pavia, Italy; 3 Unit of Nephrology, Dialysis and Transplantation, IRCCS Policlinico San Matteo Foundation, 27100 Pavia, Italy; ma.grignano@smatteo.pv.it (M.A.G.); marilena.gregorini@unipv.it (M.G.); t.rampino@smatteo.pv.it (T.R.); 4 Department of Internal Medicine and Medical Therapeutics, University of Pavia, 27100 Pavia, Italy; 5 Department of Biology and Biotechnologies “L. Spallanzani”, University of Pavia, 27100 Pavia, Italy

**Keywords:** human neutrophil elastase, HNE inhibitors, neutrophilic inflammation, protease–antiprotease imbalance, ARDS, COPD, drug discovery

## Abstract

Human neutrophil elastase (HNE) is a central mediator of neutrophil-driven inflammation. Yet, despite decades of research and drug development, therapies targeting HNE have not consistently translated into clear clinical benefits. We suggest that this translational gap partly arises from how HNE has traditionally been conceptualized, as a single enzyme to inhibit. In biological systems, however, HNE operates within a complex and tightly regulated network of proteases and inflammatory mediators. This network is spatially compartmentalized and strongly influenced by local redox conditions, making HNE activity highly context-dependent. From a systems perspective, HNE acts as an amplifier of inflammation. Its extracellular activity connects several pathological processes, including activation of innate immunity, extracellular matrix degradation, disruption of epithelial and endothelial barriers, and the transition toward chronic inflammation. In this review, we integrate insights from enzymology, systems biology, and clinical research to reassess the development of HNE inhibitors, ranging from endogenous antiproteases to more recent reversible synthetic compounds. Despite their chemical and pharmacological diversity, many of these strategies have encountered similar limitations. We therefore argue that future therapeutic approaches should move beyond the inhibition of HNE as an isolated target and instead aim to modulate the broader protease network, with particular attention to drug–target kinetics and precise delivery to disease-relevant microenvironments.

## 1. Introduction

Inflammation represents an essential host defense program triggered by infection or tissue injury and orchestrated through coordinated interactions among innate and adaptive immune cells, soluble mediators such as cytokines and proteases, and local tissue responses. In a balanced setting, the inflammatory cascade subsides once the initiating stimulus has been neutralized, allowing restoration of tissue integrity and functional homeostasis. However, when self-amplifying pathways predominate, particularly those driven by activated neutrophils and their proteolytic machinery, the response can escalate into a dysregulated state described as a “protein storm” [1]. This phenomenon parallels the concept of a cytokine storm and involves uncontrolled protease activity, oxidative mediators, extracellular matrix degradation, and secondary waves of inflammatory signaling [1].

Among the molecules central to this process, human neutrophil elastase (HNE) occupies a pivotal position in both antimicrobial defense and tissue remodeling. During normal immune function, neutrophils confine HNE within phagolysosomal compartments to digest engulfed pathogens and modulate extracellular matrix components. In pathological contexts, however, excessive or misdirected extracellular release of the enzyme becomes harmful. Crucially, HNE is also externalized via neutrophil extracellular traps (NETs), where it remains bound to the DNA scaffold, preserving its proteolytic activity and evading endogenous inhibitors. Once outside the cell, HNE degrades structural proteins including elastin, collagen, and basement membrane constituents, contributing directly to tissue injury [2]. In addition, it compromises epithelial and endothelial barrier integrity and activates pro-inflammatory signaling pathways, thereby reinforcing inflammatory amplification and promoting chronic damage [2]. These observations have positioned HNE as an attractive therapeutic target in diseases characterized by persistent neutrophilic inflammation, including chronic obstructive pulmonary disease (COPD), cystic fibrosis (CF), acute respiratory distress syndrome (ARDS), and certain autoimmune or neutrophilic dermatoses [3]. The rationale for inhibiting HNE originated from early investigations into α1-antitrypsin (AAT) deficiency, in which unopposed elastase activity drives progressive pulmonary emphysema [4]. These findings established the protease–antiprotease equilibrium as a critical determinant of lung tissue preservation. Initial therapeutic strategies therefore focused on augmenting endogenous inhibitors, including AAT replacement and the administration of naturally occurring antiproteases such as secretory leukoprotease inhibitor (SLPI) and elafin [5]. More broadly, tissue stability depends on a tightly regulated balance between neutrophil-derived proteases and their endogenous antagonists, enabling effective pathogen clearance and controlled matrix remodeling without excessive collateral injury. Disruption of this equilibrium, whether through genetic deficiency, exaggerated neutrophil activation, or oxidative inactivation of antiproteases, permits unchecked HNE activity, which in turn sustains inflammation, accelerates tissue destruction, and drives disease progression [6].

Despite decades of investigation, the development of clinically successful natural HNE inhibitors has proven difficult. Achieving sufficient selectivity, metabolic stability, and an appropriate degree of inhibition remains challenging, as many compounds also affect related serine proteases, producing unintended immunological consequences [7,8]. Pharmacokinetic barriers further complicate translation, particularly the need to deliver adequate drug concentrations to inflamed pulmonary tissues while avoiding rapid degradation. Moreover, excessive suppression of elastase activity carries the risk of impairing host defense and tissue repair mechanisms. To overcome these limitations, efforts shifted toward synthetic inhibitors beginning in the late 1980s and early 1990s, resulting in several families of potent and selective molecules [9]. Early pharmacological studies in experimental lung injury models, reported by Diane Amy Trainor, suggested that such compounds possessed the characteristics necessary to rigorously evaluate the protease–antiprotease hypothesis in clinical settings [9].

Over the ensuing decades, global research initiatives have continued to pursue synthetic HNE inhibitors capable of preventing elastase-mediated tissue destruction while maintaining acceptable safety profiles, with the aim of improving outcomes in neutrophil-driven inflammatory diseases. Multiple authoritative reviews have catalogued inhibitor chemotypes, structural determinants, and mechanisms of enzyme interaction, providing important insights into structure–activity relationships and early discovery strategies [10,11,12]. Rather than reiterating these classifications, the present review adopts a complementary perspective centred on inflammatory system dysregulation, kinetic behavior, and clinical applicability. We focus specifically on reversible synthetic inhibitors, examining concepts such as enzyme residence time, spatially protected HNE pools, and the constraints of endogenous antiprotease control during intense neutrophilic responses. By integrating pathophysiological frameworks, including protease-dominated inflammatory amplification, compartmentalized enzyme activity, and disease-specific therapeutic demands, this review aims to clarify why many highly potent inhibitors have failed to translate clinically and to identify design principles that distinguish modern drug-like candidates from earlier generations. In this context, HNE inhibition is considered not merely as enzymatic blockade, but as a strategy to modulate protease-driven inflammatory escalation in both pulmonary and systemic disorders.

## 2. Description of the Work and Literature Search Strategy

This review is organized to provide a mechanistically oriented analysis of HNE inhibition, beginning with the biological role of HNE in inflammatory diseases and the limitations of endogenous inhibitory mechanisms, followed by an overview of natural inhibitors and a comprehensive evaluation of synthetic compounds. Particular emphasis is placed on structure–activity relationships, pharmacological and kinetic properties, and clinical translatability. The final sections address current challenges, emerging strategies, and future therapeutic perspectives.

A literature search was conducted using PubMed/MEDLINE, ScienceDirect, Wiley Online Library and Nature Publishing Group to identify relevant publications from the 1990s to 2025. Search terms included combinations of “neutrophil elastase inhibitors,” “serine protease inhibitors,” “protease–antiprotease balance,” “sivelestat,” “alvelestat,” and some disease-related terms such as “ARDS” and “COPD”. Reference lists of selected articles were also screened to identify additional relevant studies. The search yielded approximately 600 records, of which 420 were screened by abstract after removal of duplicates. Full texts were evaluated for 200 articles, and approximately 120 publications were analyzed based on relevance and scientific quality. Inclusion criteria comprised original research, clinical studies, and authoritative reviews addressing HNE biology, inhibition, or therapeutic targeting. Studies not directly related to HNE, lacking sufficient methodological detail, duplicate reports, or non-English publications were excluded. As this is a narrative review, final study selection was guided by relevance to mechanistic and translational themes.

## 3. Neutrophils, Elastase and the “Protein Storm”

Building on the central role of neutrophil-derived proteases in inflammatory amplification, it is important to consider how neutrophil effector mechanisms contribute to both host protection and tissue injury. Neutrophils constitute a rapid-response component of innate immunity, migrating efficiently to sites of infection or tissue damage. Upon activation, neutrophils release granule-derived enzymes and generate reactive oxygen species (ROS). Beyond traditional activation, under specific stimuli, neutrophils can also undergo NETosis. This process is critically dependent on the translocation of HNE to the nucleus, where it cleaves histones and triggers chromatin decondensation [13]. This leads to the release of NETs. These DNA-histone scaffolds do more than just immobilize pathogens; they serve as a platform for embedded HNE, which retains its proteolytic activity while shielded from external inhibitors [13,14,15,16]. This localization fuels the aggressive ‘protein storm’ that characterizes many inflammatory states. While these mechanisms are vital for host defense, their dysregulation leads to the collateral tissue injury observed in cardiovascular, autoimmune, and malignant pathologies.

In this context, sustained protease activity becomes a key driver of structural damage and inflammatory propagation. HNE exhibits broad substrate specificity, enabling degradation of extracellular matrix proteins such as elastin, collagen, fibronectin, and proteoglycans, as well as cleavage of cell-surface receptors and activation of latent cytokines or growth factors [2]. It has been shown that high concentrations of HNE released by polymorphonuclear leukocytes in cystic fibrosis airways can transiently cleave key surface markers (CD4 and CD8) on T lymphocytes, thereby impairing their function [17]. These findings indicate that neutrophil-driven protease activity not only contributes to tissue damage but also temporarily suppresses adaptive immune responses, modulating inflammation in highly inflamed lung environments. Consistently, Domon et al. [18] reported that elevated HNE levels in cystic fibrosis airways induce cleavage of CD4 and CD8 on T cells, leading to a transient reduction in T-cell function and highlighting the immunomodulatory role of neutrophil-derived proteases in chronic lung inflammation.

When regulatory mechanisms fail, excessive elastase activity promotes progressive disruption of tissue architecture and host defence pathways, contributing to airway obstruction and structural destruction in chronic inflammatory lung diseases [19,20]. Importantly, the consequences of HNE activity extend beyond direct proteolysis. Matrix degradation products, including elastin fragments, collagen peptides, and basement membrane components, can function as damage-associated molecular patterns (DAMPs), stimulating leukocyte recruitment, macrophage activation, and downstream cytokine production [21]. Of equal importance is the capacity of NET-bound HNE to facilitate the breakdown of immune tolerance by generating and presenting modified antigens to the adaptive immune system, a mechanism distinct from simple soluble proteolysis [22]. Concurrent oxidative stress further modifies proteins through lipid peroxidation–derived aldehydes such as 4-hydroxy-2-nonenal (4-HNE), which forms covalent adducts with nucleophilic amino acid residues and contributes to cellular dysfunction, metabolic disturbance, and chronic inflammation [23].

**Figure 1:**
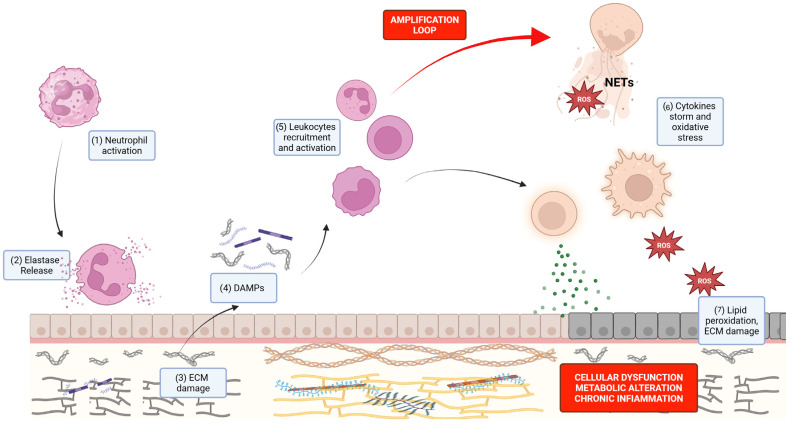
Schematic view of the sequence of events that characterize the protein storm. (1) Tissue damage or infection activates neutrophils, leading to (2) degranulation and the release of proteolytic enzymes, including neutrophil elastase (HNE). (3) HNE promotes progressive degradation of the extracellular matrix (ECM), leading to the release of structural components such as collagen, elastin, and proteoglycans into the extracellular space, where (4) they act as damage-associated molecular patterns (DAMPs), (5) activating and recruiting immune cells. This includes a positive feedback loop of further neutrophil recruitment and activation, leading to NETosis with release of reactive oxygen species (ROS) and neutrophil extracellular traps (NETs), as well as monocyte differentiation into macrophages with ROS production, and lymphocyte activation with cytokine release. (6) The sustained activation of these immune pathways culminates in a cytokine storm and increased oxidative stress. (7) Ultimately, these processes exacerbate ECM destruction and promote lipid peroxidation of cellular membranes, resulting in cellular dysfunction, metabolic imbalance, and the establishment of chronic inflammation. Created in BioRender. Rampino, T. (2026) https://BioRender.com/bvt5wdc.

Although elastase can participate in the clearance of damaged proteins, this capacity is frequently insufficient during sustained inflammation, allowing accumulation of modified macromolecules and further tissue injury. Through these interconnected mechanisms, HNE links neutrophil activation to proteolytic damage, DAMP generation, and propagation of chronic inflammatory responses. Clinically, elevated extracellular elastase activity is a defining feature of several pulmonary disorders, including COPD, CF, and bronchiectasis, where matrix degradation promotes mucus hypersecretion and impairs airway clearance [24,25,26]. Increased HNE activity also correlates with worse outcomes in ARDS, contributing to barrier disruption, edema formation, and secondary injury [26]. Beyond the respiratory system, elastase released within inflamed joints degrades cartilage components and facilitates tissue invasion in rheumatoid arthritis [27], while in dermatological conditions such as psoriasis it promotes keratinocyte proliferation through epidermal growth factor receptor (EGFR) signaling and can delay wound repair by altering matrix remodeling processes [28]. Emerging evidence further suggests a role in tumor progression and metastasis, likely mediated by extracellular matrix degradation and generation of bioactive fragments [29]. The sequence of events underlying the protein storm is reported in Figure 1.

## 4. Factors Contributing to Inadequate Inhibition of HNE by Endogenous Inhibitors

There are several reasons why endogenous inhibitors of HNE may fail to adequately control its activity. Beyond the excessive local release of HNE during intense inflammatory responses, endogenous inhibition is often compromised by poor spatial colocalization with active enzyme [30,31]. HNE can bind to cell membranes, extracellular matrix components, and bacterial surfaces, and when it is membrane-associated or matrix-bound, it may be sterically shielded from soluble inhibitors such as AAT. Although SLPI and elafin are locally expressed, they may not access all microenvironments in which HNE is active. Consequently, elastolytic activity can persist despite apparently sufficient systemic levels of endogenous inhibitors. Furthermore, ROS generated by neutrophils and macrophages at inflammatory sites can chemically modify endogenous inhibitors. AAT in fact is particularly susceptible to oxidative damage, especially at methionine residues within its reactive center loop (RCL), which markedly reduces its affinity for HNE [32]. Similarly, SLPI and elafin are vulnerable to both oxidative modification and proteolytic degradation.

Thus, inflammation simultaneously promotes HNE release while impairing the function of its natural inhibitors. In addition, endogenous inhibitors are themselves substrates for proteolytic enzymes. HNE and other proteases, including cathepsins and metalloproteinases, can cleave SLPI and elafin, rendering them inactive. This loss of inhibitory capacity establishes a self-amplifying cycle in which elastase activity further diminishes its own regulation. The efficacy of endogenous inhibition is also limited by restricted tissue penetration. Being AAT a relatively large protein, it penetrates poorly into thick mucus, necrotic tissue, or biofilm-rich environments and is therefore inefficient at reaching intracellular or pericellular sites where HNE remains active [33,34]. This limitation is particularly evident in diseases such as cystic fibrosis and chronic bronchitis, where viscous secretions hinder inhibitor diffusion.

Finally, in chronic inflammatory conditions, endogenous inhibitors are subjected to sustained demand. Persistent neutrophil recruitment maintains continuous elastase release, while inhibitor synthesis cannot indefinitely compensate for this burden. As a result, prolonged exposure to unregulated HNE activity leads to cumulative tissue damage despite the presence of regulatory mechanisms, explaining why endogenous inhibition is especially ineffective in chronic inflammatory disorders. In summary, insufficient control of HNE activity results in degradation of elastin and collagen, disruption of epithelial and endothelial barriers, amplification of inflammatory signaling, and impaired tissue repair and remodeling. These processes directly contribute to disease progression in pulmonary, cardiovascular, and systemic inflammatory conditions.

## 5. Why Has Nature-Inspired Inhibition Not Solved the HNE Problem?

Although a broad array of naturally occurring substances has been reported to attenuate HNE activity, their development trajectory reveals a recurring pattern: biochemical promise rarely translates into durable clinical benefit. Rather than a lack of potency, the central issue appears to be contextual performance, how these molecules behave within the dynamic, protease-rich, and oxidatively stressed environments in which HNE drives pathology. Natural HNE modulators span chemically and biologically diverse space, from small secondary metabolites (terpenoids, tannins, flavonoids, and other polyphenols) to macromolecular inhibitors such as proteins and peptides isolated from plants, invertebrates, and microorganisms [12]. Many demonstrate measurable in vitro inhibition and are often characterized by low intrinsic cytotoxicity and acceptable aqueous solubility. Yet these favorable attributes do not necessarily confer therapeutic viability. Rapid metabolic turnover limited epithelial permeability, instability in inflamed tissues, and inefficient accumulation at sites of neutrophil degranulation collectively restrict effective target engagement in vivo. In this regard, the disconnect between enzyme inhibition under controlled assay conditions and functional suppression of tissue-destructive elastolysis in patients remains substantial.

Plant-derived phenolics illustrate this dichotomy particularly well. Their anti-elastase activity frequently coexists with antioxidant and immunomodulatory effects, complicating mechanistic attribution and dose optimization [35,36,37,38,39,40]. Rather than acting as dedicated elastase antagonists, many function as multi-target redox-active agents whose indirect modulation of inflammatory signaling may overshadow direct catalytic inhibition. Consequently, therapeutic interpretation becomes confounded: reductions in inflammatory markers do not necessarily equate to sustained control of elastase-mediated matrix degradation. More structurally complex biological matrices, such as insect-derived extracts or venom peptides, highlight the evolutionary ingenuity of elastase regulation in nature [41,42,43]. However, their structural complexity, immunogenic potential, manufacturing challenges, and pharmacokinetic unpredictability limit scalability and regulatory feasibility. These agents often serve better as mechanistic templates than as drug candidates in their native form.

Protein-based inhibitors represent a more clinically established paradigm, exemplified by augmentation strategies using AAT. Inhaled formulations of recombinant or plasma-derived AAT aim to restore the protease–antiprotease equilibrium within the lung [44,45]. Nevertheless, practical constraints, including high dosing requirements, prolonged inhalation times, variable pulmonary deposition, and susceptibility to oxidative inactivation, restrict their efficiency [46]. Moreover, the broader clinical impact of systemic AAT supplementation remains debated, as improvements in biochemical endpoints do not always translate into consistent long-term disease modification [47,48,49]. Beyond molecule-specific challenges, translational barriers are amplified by trial design limitations [50]. Reliable surrogate biomarkers for elastase activity in vivo are lacking, necessitating prolonged and resource-intensive clinical studies to detect structural or functional endpoints. This slows iterative optimization and obscures early signals of therapeutic efficacy.

In addition, HNE is actively externalized onto NETs, where it operates within specialized microenvironments, including membrane-bound compartments and oxidatively modified matrices, that many natural inhibitors never evolved to access or withstand.

Taken together, the experience with naturally derived HNE inhibitors suggests that future progress will depend less on identifying new sources of biochemical inhibition and more on engineering context-resilient modulators. Ideal next-generation agents must retain activity under oxidative stress, penetrate relevant tissue compartments, selectively target active enzyme pools, and achieve controllable pharmacokinetics. A critical reassessment of the limitations inherent to natural scaffolds therefore provides not merely a historical overview, but a strategic blueprint for rational design of more durable elastase-directed therapies. Key distinctions among endogenous, natural and synthetic HNE inhibitors are summarized in Table 1.

## 6. Synthetic Inhibitors of HNE as Drug Candidates: Definition and General Concepts

In contrast to naturally derived peptides and proteins, synthetic inhibitors of HNE are typically rationally designed small molecules encompassing a wide range of chemical scaffolds [51,52]. Their reduced molecular size and tunable structural features enable efficient access to the catalytic pocket of HNE and direct suppression of its proteolytic activity. Compared with large protein-based inhibitors, small-molecule HNE inhibitors generally exhibit more favorable pharmacokinetic and pharmaceutical properties, including improved bioavailability, lower immunogenicity, and reduced risk of systemic toxicity. However, in the context of elastase-targeted drug discovery, maximal inhibitory potency alone is insufficient to ensure therapeutic success. The defining requirement for synthetic HNE inhibitors is a high degree of selectivity for HNE over other members of the serine protease family [53]. This challenge is particularly acute given the close structural and functional similarity between HNE and related proteases such as trypsin, chymotrypsin, proteinase 3, cathepsin G, and several key enzymes of the coagulation cascade [54,55]. Even modest cross-inhibition can disrupt essential physiological processes and severely limit clinical utility [55]. Importantly, HNE displays a relatively restricted expression pattern, being predominantly localized to neutrophils and released at sites of inflammation. Selective inhibition therefore offers the opportunity to attenuate pathological proteolysis while preserving host defense mechanisms and normal tissue homeostasis. Conversely, insufficient selectivity can lead to profound off-target effects, including bleeding complications, immune dysregulation, and impaired wound healing, outcomes that have contributed to the clinical failure of multiple elastase inhibitors.

## 7. First-Wave Synthetic Strategies Targeting HNE

The earliest attempts to attenuate excessive activity of HNE centered on small, synthetically tractable molecules engineered to interact directly with the catalytic machinery of the enzyme. An influential survey by Edwards and Bernstein over thirty years ago [56] catalogued the diverse chemotypes pursued during this formative period, reflecting the exploratory nature of inhibitor design at the time. Both peptide-derived and non-peptidic scaffolds were investigated, including chloromethyl ketones, peptidyl aldehydes and ketones, as well as a range of heterocyclic and non-heterocyclic small molecules. Among these, peptide-based transition-state mimics and short oligopeptides demonstrated particularly high potency, frequently achieving nanomolar inhibition and validating structure-activity relationship-driven optimization as a viable strategy [57]. Building on these findings, subsequent medicinal chemistry efforts focused on enhancing selectivity and improving drug-like characteristics. For example, incorporation of α-ketobenzoxazole motifs into peptidyl backbones was explored as a means to increase metabolic resilience and oral exposure [58], while Cregge and colleagues introduced pentafluoroethyl ketone functionalities that conferred measurable oral efficacy [59]. Crystallographic analyses of optimized leads confirmed productive orientation within the HNE active site and stable enzyme–inhibitor complex formation. Additional peptide-oriented designs included carboxylate-containing transition-state analogues described by Sato and co-workers [60], where the strategic inclusion of a valyl residue was instrumental in balancing inhibitory potency with reduced toxicity. Concurrently, several non-peptidic series were advanced. Imaki and collaborators reported pivaloyloxybenzene-based compounds with notable selectivity, and certain sulfonanilide derivatives demonstrated intravenous activity in preclinical models [61].

Separately, Ohmoto’s group developed desamino 5-amino-2-phenylpyrimidin-6-one derivatives equipped with an α-keto-1,3,4-oxadiazole electrophile capable of covalently engaging the catalytic Ser195 residue [62]. Despite encouraging biochemical potency and mechanistic validation, most early synthetic inhibitors did not advance beyond preclinical stages. Limitations commonly arose from inadequate discrimination against related serine proteases, unfavorable pharmacokinetic behavior, including limited oral bioavailability and rapid systemic clearance, and safety concerns linked to highly reactive warheads. Collectively, these obstacles curtailed clinical translation and highlighted the necessity for more refined design principles in subsequent generations of HNE inhibitors.

## 8. Emergence of Modern Synthetic HNE Inhibitors

**Figure 2:**
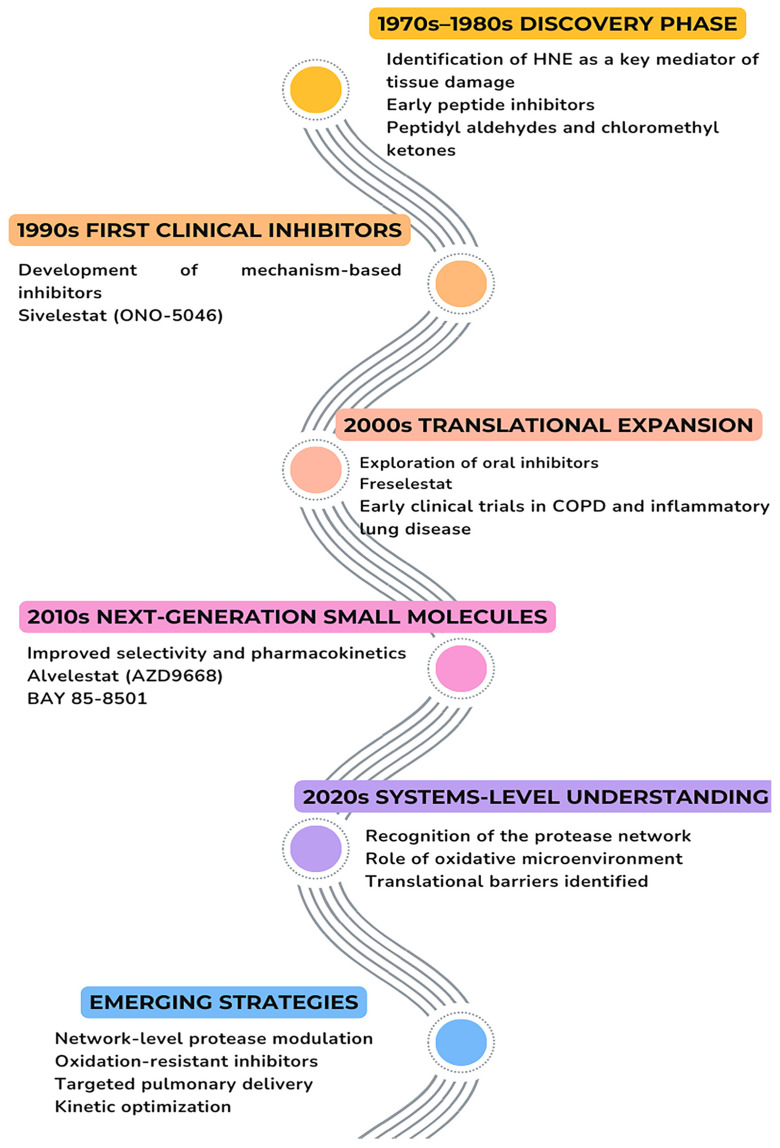
Roadmap of major milestones in HNE inhibitor discovery over time.

Recognition of the limitations inherent to early HNE inhibitors catalyzed a shift toward more sophisticated design strategies, prioritizing clinical translatability alongside enzymatic potency. Rather than focusing exclusively on maximal inhibition, modern approaches emphasized selectivity, improved pharmacokinetic behavior, and reduced toxicity. This transition was enabled by advances in high-resolution HNE crystal structures, which facilitated structure-based drug design and more precise optimization of active-site interactions [63,64,65]. Within this evolving landscape, sivelestat represents a pivotal example of a rationally designed HNE inhibitor [63,64,65]. Although developed earlier than many contemporary agents, it embodies key principles, such as balanced potency and improved drug-like properties, that continue to inform current small-molecule inhibitor development. As such, sivelestat occupies an intermediate position between early empirical inhibitors and later generations of elastase-targeted therapeutics, serving as a conceptual bridge toward modern HNE inhibitor design. A scheme showing the evolution of HNE inhibitors is shown in Figure 2.

## 9. HNE Inhibitors and the Protease Inhibition Paradox: Success in Chronic Disease, Struggle in Acute Critical Care

Sivelestat is the first clinically approved inhibitor of HNE [66,67] and represents a pivotal case study in protease-targeted therapy. Its development highlights a broader paradox: while protease inhibition has transformed outcomes in chronic viral diseases such as HIV and hepatitis C, translation in acute critical care syndromes like ARDS has been inconsistent. Despite strong mechanistic rationale and encouraging early results, sivelestat achieved only regional approval and limited global adoption. This divergence reflects a fundamental challenge in ARDS therapeutics, the disconnect between molecular target engagement and meaningful clinical benefit in biologically heterogeneous syndromes [68,69,70,71].

### 9.1. Protease Inhibition in Chronic Diseases vs. Acute Critical Care

Chronic viral infections rely on essential, non-redundant proteases with stable expression and clearly defined causal roles. Sustained inhibition directly disrupts replication, and therapeutic effects are predictable. Similarly, in some chronic inflammatory disorders, protease activity acts as a persistent and central disease driver, allowing prolonged dosing to maintain effective suppression. In contrast, ARDS is a rapidly evolving syndrome characterized by network-level inflammation, interpatient heterogeneity, and functional redundancy among proteases such as cathepsins, matrix metalloproteinases, and proteinase 3 [72,73,74]. Within this adaptive system, elastase contributes to tissue injury but is not universally dominant. Compensatory pathways may blunt the impact of selective inhibition, and the therapeutic window is narrow and highly time-dependent. Consequently, although sivelestat effectively inhibited HNE, modulation of a single enzymatic node was often insufficient to alter the broader inflammatory trajectory.

### 9.2. The Temporal and Stratification Mismatch

In chronic disease, protease inhibitors are typically introduced before irreversible tissue damage occurs. In ARDS, treatment often begins after substantial alveolar injury is established [75,76]. Elastase inhibition may therefore limit further damage without reversing structural destruction. Sivelestat’s short half-life and need for continuous infusion further illustrate the challenge of aligning pharmacokinetics with the brief period of protease-driven injury. Patient selection represents an additional constraint. Chronic therapies target individuals with confirmed molecular pathology, whereas ARDS trials have relied primarily on clinical criteria rather than biomarkers of neutrophil-predominant inflammation [77,78,79,80]. The absence of stratification likely diluted potential benefits in elastase-dominant subgroups.

### 9.3. Reinterpreting the Sivelestat Experience

Rather than reflecting simple inefficacy, sivelestat illustrates structural barriers to protease targeting in acute critical illness: biological heterogeneity, protease redundancy, narrow therapeutic windows, and limited biomarker guidance. Its trajectory underscores the need for early intervention, endotype stratification, and potentially combination approaches addressing parallel inflammatory pathways. Sivelestat therefore represents a proof of pharmacological feasibility coupled with a translational lesson: selective HNE inhibition is achievable, but clinical success depends on precise alignment between drug properties, disease phase, and patient biology.

## 10. From First-Generation Lessons to Next-Generation HNE Inhibitors: Overcoming Early Limitations Through Transitional Chemical Space and Rational Small-Molecule Optimization

The clinical and pharmacological experience with sivelestat fundamentally reframed elastase inhibitor development, revealing that the primary limitation was not insufficient potency but a lack of contextual alignment between inhibitor design and disease biology. Early compounds highlighted key constraints, including suboptimal pharmacokinetics, limited tissue exposure, protease-network redundancy, and the absence of biomarker-guided patient stratification, shifting the field away from a purely potency-driven approach toward an integrated strategy encompassing drug kinetics, delivery, and disease context.

These insights, rather than being invalidated, refined the therapeutic rationale for targeting HNE, and guided subsequent medicinal chemistry efforts toward improving metabolic stability, reversibility, selectivity, and suitability for chronic administration. Within this evolutionary trajectory, sivelestat (and alvelestat) emerged as pivotal transitional molecules bridging early reactive inhibitors and later structure-guided small molecules. Their design marked a shift toward exploiting the physicochemical architecture of the HNE catalytic pocket through optimized non-covalent interactions, often incorporating sulfonyl-containing motifs and polar substituents to enable reversible yet high-affinity binding. This approach demonstrated that effective elastase inhibition could be achieved without permanent enzyme inactivation, establishing reversibility as a viable therapeutic paradigm while improving selectivity over related serine proteases. At the same time, these scaffolds underscored the importance of balancing enzymatic potency with pharmacokinetic and safety profiles, as well as the challenges of translating biochemical inhibition into clinical benefit. Although their inhibitory potency remained modest compared with later agents, they provided critical structural and conceptual foundations for rational optimization, ultimately enabling the development of second-generation inhibitors with enhanced selectivity, drug-like properties, and translational potential.

The experience with sivelestat reframed elastase inhibitor development. The challenge was not insufficient potency, but insufficient contextual fit between inhibitor design and disease biology. Early compounds exposed key constraints, including pharmacokinetic limitations, incomplete tissue exposure, protease-network redundancy, and lack of biomarker-guided deployment. These insights shifted the field from a potency-driven model toward an integrated strategy incorporating drug kinetics, delivery, and disease stratification. Importantly, they did not invalidate the therapeutic rationale for HNE targeting; rather, they clarified the requirements for achieving clinical impact. Subsequent medicinal chemistry efforts therefore focused on improving stability, reversibility, selectivity, and suitability for chronic administration. The emergence of second-generation small molecules reflects this conceptual evolution, from proof-of-concept inhibitors to structurally refined agents designed for sustained and context-appropriate modulation of HNE activity.

### 10.1. Second-Generation Refinement: The Case of Alvelestat

The development of alvelestat (AZD9668) reflects a deliberate evolution in elastase inhibitor design based on lessons learned from earlier agents [81]. Rather than refining the same reactive pharmacology, alvelestat represents a strategic pivot toward chemical stability, reversibility, and suitability for chronic administration. Conceptually, it can be viewed as an attempt to reconcile potent neutrophil elastase inhibition with the pharmacological demands of long-term inflammatory disease management. Its scaffold departs from ester-based, hydrolytically labile inhibitors and adopts a chemically robust, non-peptidic architecture that enhances metabolic stability while preserving high affinity for the elastase active site. Multiple heteroaromatic elements create a rigid molecular framework supporting defined interactions within the catalytic pocket. Structural features such as the trifluoromethyl substituent contribute to pharmacokinetic stability, while the methylsulfonyl group promotes precise engagement with catalytic and adjacent residues. Collectively, these modifications represent a move toward structural durability and optimized drug-like properties. Mechanistically, alvelestat acts as a fully reversible competitive inhibitor that binds non-covalently within the catalytic pocket containing Ser195, preventing substrate access without forming a covalent intermediate. Kinetic analyses indicate a slow-binding mechanism characterized by rapid formation of an initial enzyme–inhibitor complex followed by conformational stabilization into a more tightly bound state, resulting in prolonged residence time while maintaining reversibility [82]. Enzymatic activity is restored upon dilution or inhibitor removal, confirming the absence of permanent inactivation. Alvelestat advanced into phase II clinical trials for chronic respiratory conditions, including COPD, bronchiectasis, and AATD [83,84]. Although improvements in lung function endpoints were not consistently demonstrated, the compound exemplifies modern HNE inhibitor design, combining selectivity, oral bioavailability, and suitability for prolonged administration.

### 10.2. High-Affinity Optimization and Clinical Advancement: BAY 85-8501

Further refinement of non-covalent inhibitor design led to the development of BAY 85-8501, a highly potent and selective reversible inhibitor intended for inflammatory lung diseases [85]. Preclinical studies demonstrated picomolar inhibitory potency together with strong selectivity over related serine proteases, indicating highly efficient target engagement compared with earlier molecules that typically exhibited nanomolar activity. The compound also displayed pharmacokinetic properties compatible with oral administration, reinforcing the feasibility of systemic delivery for elastase inhibition. In clinical evaluation involving patients with non-cystic fibrosis bronchiectasis, oral administration over several weeks was generally well tolerated, with mostly mild to moderate adverse events [86]. Despite its favorable biochemical profile, however, clinical studies did not demonstrate significant improvements in key functional outcomes over short treatment durations [86]. These findings highlighted an important translational challenge: achieving high enzymatic inhibition does not necessarily translate into measurable clinical benefit, particularly in complex inflammatory diseases where drug exposure at the site of pathology, disease heterogeneity, and compensatory biological mechanisms may influence therapeutic response.

## 11. Translational Lessons and Pharmacological Differentiation Across Small-Molecule HNE Inhibitors

Comparative evaluation of these small-molecule inhibitors reveals several recurring themes that extend beyond individual compounds. First, the relationship between systemic pharmacokinetics and local airway exposure appears critical. Oral administration may not consistently achieve sufficient concentrations within airway secretions to neutralize extracellular elastase activity, particularly in diseases characterized by thick mucus and impaired drug penetration. Second, selective targeting of HNE alone may not fully address the broader protease-antiprotease imbalance present in chronic inflammatory lung disorders. Endogenous antiproteases and multiple proteolytic enzymes contribute to tissue injury, suggesting that narrow inhibition of a single protease may yield limited clinical impact unless combined with additional therapeutic strategies. Third, clinical endpoints such as lung function or symptom scores may require longer treatment durations or earlier intervention stages to demonstrate benefit. These considerations collectively emphasize that biochemical potency, while essential, is insufficient on its own to ensure clinical success. The experience with transitional and advanced small-molecule inhibitors therefore provided critical insights into pharmacodynamic requirements, delivery challenges, and disease-specific factors that must be addressed in future therapeutic development.

## 12. Scaffold Diversification and Medicinal Chemistry Expansion in HNE Inhibitor Development

Recognition of the limitations associated with early inhibitors stimulated extensive medicinal chemistry efforts aimed at scaffold diversification and optimization. Derivative programs originating from sivelestat-related chemistry explored modifications intended to improve metabolic stability, pharmacokinetic behavior, and suitability for chronic administration. One representative outcome of these efforts was the identification of freselestat (ONO-6818), which, although emerging from the same developmental lineage, incorporated a structurally distinct core architecture. This compound demonstrated oral activity together with high affinity and selectivity for HNE in preclinical studies, illustrating the potential of scaffold redesign to enhance pharmacological properties [87,88]. However, clinical development was discontinued because of safety and tolerability concerns observed during longer-term administration, including elevations in liver function parameters. These findings underscore the importance of balancing potency with systemic safety, particularly for chronic indications requiring sustained exposure.

In an interesting study, Crocetti et al. [89], rather than pursuing entirely novel chemotypes, adopted a hybrid design strategy, transplanting the pharmacologically validated 4-(sulfamoyl)phenyl pivalate fragment of sivelestat into diverse scaffolds. This work exemplifies a rational optimization strategy, in which a validated pharmacophore is used to guide the design of next-generation inhibitors. It reinforces the idea that progress in this field is less about discovering entirely new targets and more about refining molecular interactions, stability, and context-appropriate activity, key principles for overcoming past translational limitations.

Additional analogues derived from modifications of related scaffolds, including peptidic variants designed to improve metabolic stability and potency, have also been investigated. Although none have yet achieved clinical success beyond earlier inhibitors, these programs contributed substantially to understanding structure–activity relationships and identified key determinants governing selectivity, stability, and pharmacokinetic behavior [90,91].

## 13. Beyond Small Molecules: Peptide-Based and Biologically Inspired Inhibition Strategies

While small-molecule inhibitors dominate the therapeutic landscape, alternative approaches have explored biologically inspired strategies aimed at achieving higher specificity and more physiologically relevant inhibition. Depelestat (DX-890, EPI-hNE4) represents a peptide-based inhibitor engineered to target HNE with high selectivity [92]. This recombinant protein, composed of 56 amino acids, is structurally modelled on natural protease inhibitors and functions as a reversible inhibitor by forming stable enzyme–inhibitor complexes. Unlike low-molecular-weight compounds, its peptide framework mimics endogenous regulatory mechanisms, offering the potential for highly specific elastase neutralization. Preclinical studies and ex vivo analyses demonstrated effective inhibition of both membrane-associated and soluble elastase activity, supporting its therapeutic rationale in inflammatory lung disorders [93]. Clinical investigations progressed into early-phase trials in conditions such as acute respiratory distress syndrome and cystic fibrosis, where excessive neutrophil elastase activity contributes to tissue damage and inflammatory amplification [94]. However, peptide-based inhibitors present distinct pharmacokinetic and formulation challenges, including parenteral administration requirements, susceptibility to proteolytic degradation, and limited tissue penetration. These constraints have complicated clinical translation despite promising biological activity. Nevertheless, depelestat has provided important proof-of-concept for biologic targeting of HNE and has contributed to validating elastase inhibition as a therapeutic strategy [87]. Its development also highlights the potential value of hybrid approaches, such as peptidomimetics or engineered biologics, aimed at combining high specificity with improved stability and drug-like properties. The principal biologic and synthetic HNE inhibitors discussed in this review are summarized in Table 2.

## 14. Emerging Combination or Multi-Mechanism Strategies

Nanoparticle-based and multifunctional therapeutic approaches are emerging as promising strategies to modulate neutrophil-driven pathology by simultaneously targeting neutrophil activation, oxidative stress, and NET formation, three tightly interconnected processes that drive tissue injury in inflammatory lung diseases. Nanocarriers such as liposomes, polymeric nanoparticles, dendrimers, and biomimetic systems (e.g., cell membrane–coated nanoparticles) can be rationally engineered to enhance drug accumulation at sites of inflammation or within activated neutrophils, thereby improving local drug concentration while minimizing systemic exposure [95,96,97,98,99]. These platforms enable the co-delivery or spatially controlled release of elastase inhibitors, antioxidants such as N-acetylcysteine, and anti-inflammatory agents, which in preclinical models have been shown to reduce ROS production, suppress NET formation, and limit protease-mediated tissue damage in conditions such as acute lung injury and sepsis.

Recent work, including the study reported by Crocetti et al. [100], further highlights how advanced nanomedicine design can integrate multiple functionalities into a single platform, enabling simultaneous modulation of oxidative stress and inflammatory signalling pathways. The authors emphasize the importance of stimuli-responsive systems, such as ROS-sensitive nanoparticles, that exploit the pathological microenvironment to trigger site-specific drug release, thereby enhancing efficacy while preserving healthy tissue. In addition, the paper underscores the growing relevance of biomimetic and surface-functionalized nanoparticles capable of selectively interacting with activated neutrophils or inflamed endothelium, improving targeting precision in complex inflammatory settings.

**Figure 3:**
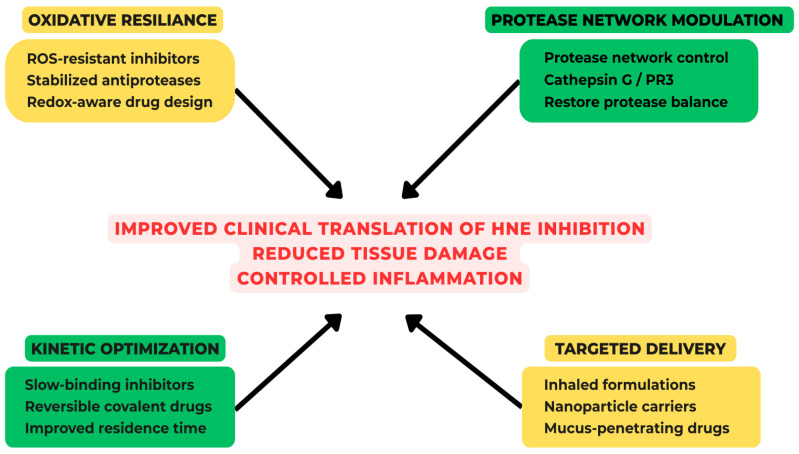
Next-generation therapeutic strategies targeting the HNE-driven proteolytic network.

Multifunctional nanoplatforms are particularly attractive because they can combine complementary therapeutic mechanisms within a single construct, for example, incorporating both ROS-scavenging capacity and protease inhibition, or coupling targeting ligands with controlled-release chemistries. Alongside these systems, enzyme-mimetic nanomaterials (“nanozymes”) exhibiting superoxide dismutase- or catalase-like activity are being actively explored to directly neutralize oxidative stress, thereby indirectly attenuating NETosis and downstream inflammatory cascades. Collectively, these advances point toward a new generation of integrated therapeutic strategies that move beyond single-target inhibition, instead addressing the complex, networked nature of neutrophil-driven pathology. The conceptual framework for next-generation strategies targeting human neutrophil elastase is reported in Figure 3.

Although these approaches remain largely preclinical, they offer a conceptual advantage over single-target drugs by addressing the complex, self-amplifying nature of neutrophil-mediated inflammation and may represent an important future direction for treating conditions such as acute respiratory distress syndrome, chronic obstructive pulmonary disease, cystic fibrosis, and bronchiectasis. It is our opinion that the future of elastase inhibition lies not in stronger inhibitors, but in smarter alignment between drug, biology, and patient. The recurring determinants of translational failure across HNE inhibitor development programs are summarized in Table 3.

## 15. Conclusions

Despite decades of mechanistic investigation and extensive medicinal chemistry efforts, the central paradox of HNE-directed therapy remains unresolved, and the question is: why has robust biochemical inhibition so rarely translated into durable clinical benefit? We contend that this disconnect reflects not simply technical shortcomings in drug design, but a deeper conceptual misalignment in how HNE has been historically framed. It is not as a dynamically regulated node embedded within a spatially compartmentalized, redox-sensitive, and functionally redundant protease–inflammatory network, but rather as an isolated enzymatic target amenable to linear pharmacological suppression. This reductionist view, while instrumental in advancing early inhibitor development, has likely obscured the complex, context-dependent roles that HNE plays across different disease states and microenvironments.

Accordingly, this review, rather than merely cataloguing inhibitor classes or reiterating structure–activity relationships, advances a systems-oriented translational framework that integrates enzyme kinetics with higher-order regulatory layers. It includes inflammatory amplification circuits, endogenous antiprotease buffering systems, cellular trafficking dynamics, and disease-specific pathophysiological contexts. This approach acknowledges that the functional impact of HNE activity is not dictated solely by its catalytic efficiency, but also by its position within a tightly interconnected and adaptable biological network. This can compensate for, amplify, or redirect proteolytic signals in response to therapeutic intervention.

If we aim to provide a more nuanced perspective on previous translational failures, it becomes necessary to reinterpret HNE inhibition as a context-dependent form of modulation, rather than a simple enzymatic blockade. This approach suggests that clinical inefficacy may arise not only from insufficient target engagement, but also from inappropriate timing, localization, or magnitude of inhibition, as well as from unanticipated network-level adaptations. This perspective further implies that successful therapeutic strategies will likely require greater precision in targeting specific disease stages, cellular compartments, or patient subpopulations, potentially in combination with approaches that modulate complementary inflammatory or proteolytic pathways. Finally, the exciting path forward lies in embracing this complexity rather than attempting to circumvent it. By integrating insights from systems biology, chemical biology, and clinical pharmacology, future efforts can move toward the rational design of next-generation inhibitors that are not only potent and selective, but also context-aware and mechanistically aligned with the dynamic biology of HNE. Such agents, guided by biomarker-driven stratification and informed by real-time measures of network activity, may be better positioned to achieve sustained therapeutic benefit. In this way, resolving the longstanding paradox of HNE-directed therapy will depend less on discovering more powerful inhibitors, and more on deploying them within a framework that accurately reflects the biological systems they are intended to modulate.

## Figures and Tables

**Table 1:** Comparison between endogenous, natural, and synthetic HNE inhibitors.

Property	Endogenous Inhibitors (AAT, SLPI, Elafin)	Natural Small-Molecule Inhibitors	Synthetic Small-Molecule Inhibitors	Peptide or Biologic Inhibitors
Molecular size	Large proteins	Small–medium	Small	Medium-sized
Selectivity for HNE	High	Variable	Tunable, often high	Very high
Oxidative stability	Poor (susceptible to ROS)	Variable	Optimizable	Moderate
Tissue penetration	Limited	Often limited	Generally good tissue penetration	Limited
Pharmacokinetics	Short half-life	Rapid metabolism	Optimizable	Limited stability
Immunogenicity risk	Low–moderate	Low	Low	Potential risk
Manufacturing complexity	High	Moderate	Moderate	High
Clinical success	Partial (AAT therapy)	Minimal	Moderate (regional approvals, clinical trials)	Limited
Main limitation	Oxidative inactivation and limited compartment access	Low potency and metabolic instability	Context-dependent efficacy and translational challenges	Delivery constraints and metabolic instability

**Table 2:** Major synthetic and biologic inhibitors of HNE.

Compound	Type	Clinical Development/Indication	Key Advantages	Main Limitations	Reference
Early peptidyl ketones/aldehydes	Peptide-derived inhibitors	Preclinical	High potency; early mechanistic validation	Poor pharmacokinetics; toxicity	[49,50,51]
**α**-ketoheterocycles/oxadiazoles	Synthetic small molecules	Preclinical	Improved selectivity compared with early inhibitors	Limited clinical translation	[52,53]
Sivelestat (ONO-5046)	Small-molecule inhibitor	Approved in Japan/Korea for ARDS; trials elsewhere	First clinically approved HNE inhibitor; proof of target validity	Short half-life; continuous infusion; limited global efficacy	[54,57,58,59,60,61,70]
Alvelestat (AZD9668)	Oral small-molecule inhibitor	Phase II trials in COPD, bronchiectasis, AAT deficiency	Oral bioavailability; high selectivity; suitable for chronic therapy	Limited improvement in lung function endpoints	[72,73,74,75]
BAY 85-8501	Potent small-molecule inhibitor	Phase IIa bronchiectasis	Very high potency and selectivity; good tolerability	Limited clinical efficacy demonstrated	[76,77]
Freselestat (ONO-6818)	Small-molecule inhibitor (sivelestat lineage)	Phase II (discontinued) in COPD, AAT deficiency	Oral activity; improved scaffold stability	Liver toxicity signals; program discontinued	[78,79]
Depelestat (DX-890/EPI-hNE4)	Recombinant peptide biologic	Phase I–II ARDS, cystic fibrosis	High specificity; mimics endogenous inhibition	Poor tissue penetration; proteolytic instability	[82,83]

**Table 3:** Key limiting factors contributing to the translational failure of HNE inhibitors.

Limiting Factor	Mechanism	Consequence	Clinical Implication
Biological redundancy	Compensatory activity of other proteases	Partial pathway suppression	Limited efficacy in complex syndromes (e.g., ARDS)
Enzyme compartmentalization	Membrane-bound, NET-associated, or matrix-bound HNE becomes inaccessible	Incomplete target engagement	Persistent elastase activity despite therapy
Oxidative microenvironment	ROS modify inhibitors and endogenous antiproteases	Reduced inhibitory capacity	Reduced effectiveness in inflamed tissues
Pharmacokinetic–pharmacodynamic mismatch	Insufficient drug concentration at disease site	Suboptimal inhibition	Failure despite potent in vitro activity
Timing of intervention	Intervention after irreversible tissue damage	Limited reversibility	Poor outcomes in acute disease
Patient heterogeneity	Variable neutrophil burden and disease endotypes	Diluted treatment effect	Negative or inconclusive trials
Biomarker limitations	Lack of validated biomarkers of elastase activity	Poor patient selection	Inefficient clinical trial design
Delivery constraints	Poor penetration into mucus or inflamed tissue	Reduced local exposure	Particularly relevant in lung diseases

## Data Availability

No new data were created or analyzed in this study. Data sharing is not applicable to this article.

## Abbreviations

The following abbreviations are used in this manuscript:

AAT	α1-antitrypsin
AATD	α1-antitrypsin deficiency
ARDS	Acute respiratory distress syndrome
CF	Cystic fibrosis
COPD	Chronic obstructive pulmonary diseases
DAMPs	Damage-associated molecular patterns
ECM	Extracellular matrix
EGFR	Epidermal growth factor receptor
HNE	Human neutrophil elastase
NETs	Neutrophil extracellular trap
ROS	Reactive oxygen species
SLPI	Secretory leukoprotease inhibitor
4-HNE	4-hydroxy-2-nonenal
